# Multicopy plasmid integration in *Komagataella phaffii* mediated by a defective auxotrophic marker

**DOI:** 10.1186/s12934-017-0715-8

**Published:** 2017-06-08

**Authors:** Maritza Ocampo Betancur, Viviane Castelo Branco Reis, André Moraes Nicola, Janice Lisboa De Marco, Lídia Maria Pepe de Moraes, Fernando Araripe Gonçalves Torres

**Affiliations:** 1 Laboratório de Biologia Molecular, Departamento de Biologia Celular, Instituto de Ciências Biológicas, Universidade de Brasília, Brasília, DF 70910-900 Brazil; 2 Faculdade de Medicina, Laboratório de Imunologia Celular, Universidade de Brasília, Brasília, DF 70910-900 Brazil

**Keywords:** *Komagataella phaffii*, Leucine biosynthesis, Auxotrophic marker, Multicopy integration, Expression system

## Abstract

**Background:**

A commonly used approach to improve recombinant protein production is to increase the levels of expression by providing extra-copies of a heterologous gene. In *Komagataella phaffii* (*Pichia pastoris*) this is usually accomplished by transforming cells with an expression vector carrying a drug-resistance marker following a screening for multicopy clones on plates with increasingly higher concentrations of an antibiotic. Alternatively, defective auxotrophic markers can be used for the same purpose. These markers are generally transcriptionally impaired genes lacking most of the promoter region. Among the defective markers commonly used in *Saccharomyces cerevisiae* is *leu2*-*d*, an allele of *LEU2* which is involved in leucine metabolism. Cells transformed with this marker can recover prototrophy when they carry multiple copies of *leu2*-*d* in order to compensate the poor transcription from this defective allele.

**Results:**

A *K. phaffii* strain auxotrophic for leucine (M12) was constructed by disrupting endogenous *LEU2*. The resulting strain was successfully transformed with a vector carrying *leu2*-*d* and an EGFP (enhanced green fluorescent protein) reporter gene. Vector copy numbers were determined from selected clones which grew to different colony sizes on transformation plates. A direct correlation was observed between colony size, number of integrated vectors and EGFP production. By using this approach we were able to isolate genetically stable clones bearing as many as 20 integrated copies of the vector and with no significant effects on cell growth.

**Conclusions:**

In this work we have successfully developed a genetic system based on a defective auxotrophic which can be applied to improve heterologous protein production in *K. phaffii*. The system comprises a *K. phaffii leu2* strain and an expression vector carrying the defective *leu2*-*d* marker which allowed the isolation of multicopy clones after a single transformation step. Because a linear correlation was observed between copy number and heterologous protein production, this system may provide a simple approach to improve recombinant protein productivity in *K. phaffii*.

**Electronic supplementary material:**

The online version of this article (doi:10.1186/s12934-017-0715-8) contains supplementary material, which is available to authorized users.

## Background

The methylotrophic yeast *Komagataella phaffii* (formerly *Pichia pastoris*) is one of the most important expression platforms for the production of recombinant proteins [1, 2]. It offers many advantages such as: easy genetic manipulation; growth at high cell densities, e.g. 200 g L^−1^ dry weight during a glucose-limited fed-batch cultivation [3]; ability to produce heterologous proteins at high levels, e.g. more than 18 g L^−1^ of lignocellulolytic enzyme *Tr*CBH2 [4]; and post-translational modifications similar to higher eukaryotes [5].

Due to its biotechnological interest, many studies have focused on the genetic improvement of *K. phaffii* in order to optimize protein production. A well-established approach to accomplish this is to assure high transcription levels of a heterologous gene thus favoring the translation of the desired mRNA. Typically, this can be achieved by constructing expression cassettes under the control of strong promoters or/and by screening clones bearing multiple copies of the desired gene (for a review see [6, 7]). Genetic strategies are available for the isolation of multicopy clones. Yeast cells can be transformed with vectors carrying extra copies of the expression cassette cloned *in tandem* (multimeric construction) [8] or successive rounds of transformation can be performed using different selection markers [9]. In both cases cloning is labor-intensive and the extent of copy number increase is limited [10].

Another option consists in the use of antibiotic-resistance markers, in which case one looks for transformants growing in higher concentrations of the antibiotic (direct selection method) [11]. A previous study showed that this type of selection resulted in the isolation of sporadic multicopy integrants with increased productivity of the desired protein [12]. Dominant markers can also give rise to multicopy clones by posttransformational vector amplification (PTVA) [13] or liquid PTVA [14]. It has been demonstrated that after transformation with a few copies of a vector carrying a drug-resistance marker, such as zeocin or G418, cells can be selected in stepwise higher concentrations of the drug resulting in the selection of multicopy clones. The use of PTVA in combination with the use of rDNA non-transcribed sequence (NTS) as an integration target sequence resulted in multicopy clones in *K. phaffii* [15]. Besides being a laborious and expensive method due to the high costs of eukaryotic antibiotics, one disadvantage of the use of dominant markers is that a significant number of clones show increased natural drug-resistance for other reasons than vector copy number.

An alternative strategy is based in the use of defective auxotrophic markers, i.e. genes that are poorly transcribed typically due to extensive deletions of their promoters. To compensate the low transcription levels, cells need to amplify the copy number of the defective marker in order to recover prototrophy. Consequently, copy number of the neighboring heterologous gene is also amplified [16]. An example of such defective marker is the *leu2*-*d* allele which contains only 29 base pairs of the original promoter and is commonly used in *S. cerevisiae* for plasmid maintenance at high copy number under selective pressure [17]. Due to this feature, this system has also been used to increase recombinant protein production in this yeast [18–20]. This prompted us to develop an analogous system to be employed in *K. phaffii*. To accomplish this, we sought the construction of a *K. phaffii* strain auxotrophic for leucine and the development of an integrative expression vector based on *leu2*-*d* as a tool to increase recombinant protein production in this yeast.

## Results and discussion

### Construction of a leu2 auxotrophic strain

Genetic manipulation of *K. phaffii* is possible due to its widely used transformation system which enables integration of foreign DNA into the genome via homologous recombination [21]. This approach has been successfully used to disrupt several genes in order to create auxotrophic mutants, e.g. *URA5* [22], *ARG1, ARG2, ARG3, HIS1, HIS2, HIS5* and *HIS6* [23]. Recently, a CRISPR-Cas9 system was developed for *K. phaffii* which has greatly facilitated gene knock out in this yeast [24].

**Fig. 1:**
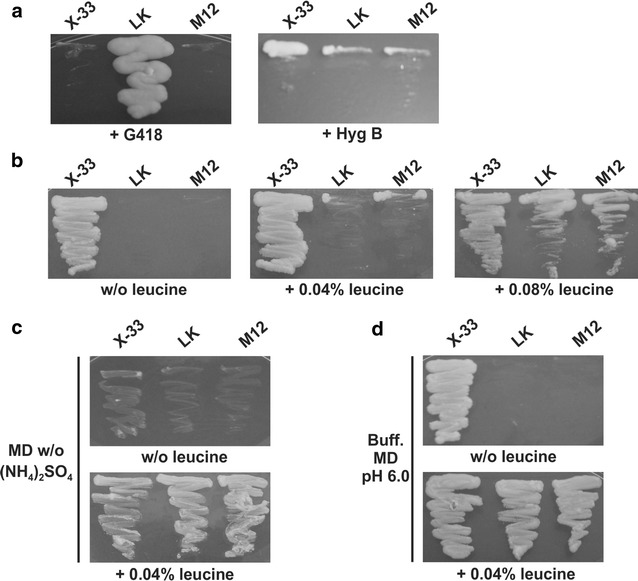
Strain phenotypic analysis. Strains LK and M12 were grown in different media to confirm drug resistance and leucine assimilation. X-33 was used as a control. **a** YPD containing G418 or hygromycin B (Hyg B). **b** MD lacking or not leucine in different concentrations. **c** MD with leucine replacing ammonium sulfate. **d** Buffered MD (pH 6.0) containing leucine. *X-33* wild-type strain with intact *LEU2* gene, *LK leu2* strain disrupted with *kan* cassette, *M12* strain obtained after marker removal with CreA recombinase

We sought the development of a leucine auxotrophic strain by gene knock out of the endogenous *K. phaffii LEU2* gene with a *leu2::kan* disruption cassette. The resulting strain (LK) was then transformed with a plasmid expressing CreA recombinase for marker removal thus generating strain M12 (see Additional file 1 for details). Growth analysis on plates containing G418 or hygromycin B confirmed the loss of all dominant markers (Fig. 1a). The phenotypic behavior of the strains obtained with respect to leucine assimilation was then analyzed. As expected, LK and M12 strains were not able to grow on MD lacking leucine (Fig. 1b). We expected that supplementation of MD medium with leucine would allow both *leu2* strains to recover prototrophy, however, even with an oversuplemmentation (0.08%) of leucine cells were unable to grow (Fig. 1b). We reasoned that ammonium sulphate present in MD medium could be affecting leucine uptake because when this salt was replaced by 0.04% leucine as sole nitrogen source both *leu2* strains grew as well as wild-type X-33 (Fig. 1c). This result is in accordance with a previous work [25] which showed that cells grown in minimal medium exhibited an increase in the rate of leucine uptake when this amino acid was the sole nitrogen source. It is known that the addition of NH_4_^+^ to yeast cells causes nitrogen catabolite inactivation and repression of several enzymes and permeases involved in the utilization of secondary nitrogen sources [26]. Leucine has been shown to be transported by at least three systems in *S. cerevisiae*: GAP (general amino acid permease), S1 (high-affinity permease) and S2 (low-affinity permease) [27]. In NH_4_^+^-containing media the activity of GAP is inhibited [28, 29] and the activity of S1 and S2 proteins is strongly reduced [30]. In addition, two redundant low affinity leucine permeases coded by the *AGP2* and *AGP3* genes are overexpressed when other permeases are inhibited [31]. The observation that prototrophy could only be achieved in high leucine concentrations can be explained by the fact that, in the presence of NH_4_^+^, leucine uptake is mainly due to low-affinity permeases. The effects of NH_4_^+^ on leucine permeases could be related to intracellular pH as it has been shown that a *S. cerevisiae leu2* strain was more sensitive to internal acidic conditions [32]. In yeast, amino acids and other nutrients are taken up by a proton symport mechanism [33]. When NH_4_^+^ (a conjugated weak acid) is internalized by specific transporters it undergoes deprotonation and as a result the proton gradient is dissipated leading to acidification of the cytosol [34]. To test this, MD medium was buffered to pH 6.0 and as a result prototrophy was readily recovered as shown in Fig. 1d.

### Heterologous expression in *K. phaffii* M12

**Fig. 2:**
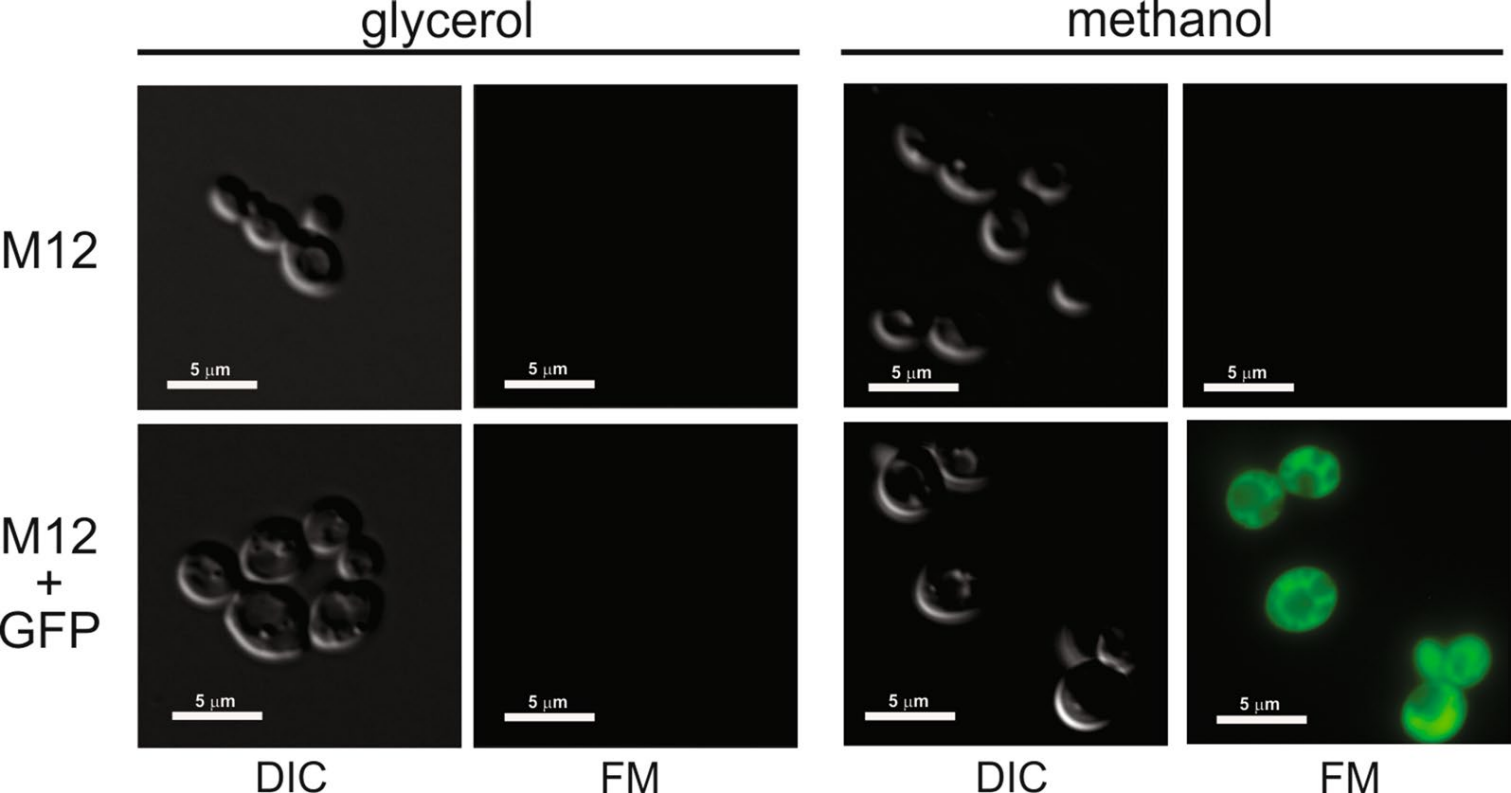
Heterologous expression in *K. phaffii* M12. Intracellular expression of enhanced green fluorescent protein (EGFP) was visualized by epifluorescence microscopy. Untransformed (M12) and transformed (M12 + GFP) cells were viewed under differential interference contrast (DIC) or fluorescence (FM) microscopy after growth in glycerol or methanol containing media. All images were collected with the same exposure time

In order to test *K. phaffii* M12 for heterologous expression we constructed an integrative vector, pGFP-L2, containing *LEU2* as selectable marker and the enhanced GFP (EGFP) reporter gene under the control of the methanol-inducible P_*AOX1*_ promoter. After electroporation of *K. phaffii* M12 with linearized pGFP-L2, one colony was randomly chosen and grown under non-inducible (glycerol) and inducible (methanol) conditions to test for intracellular production of the EGFP. Figure 2 shows the result of fluorescence microscopy analysis for the presence of EGFP. As expected, no fluorescence was detected under non-inducible conditions and EGFP could only be detected when methanol was added. This result shows that *K. phaffii* M12 can recover prototrophy upon transformation with a vector carrying a wild-type *LEU2* marker. However, since the strategy used for *LEU2* disruption in M12 left the promoter region intact, a single copy of a defective version of this gene could potently integrate at this *locus* by homologous recombination and reestablish prototrophy without the need of other copies of the marker. In order to develop a system based on defective *LEU2* for multicopy integration, we decided to use the *S. cerevisiae leu2*-*d* allele which has 68% identity with the *K. phaffii LEU2* homologue and should reduce the chances of homologous recombination at the disrupted *leu2 locus* in M12.

### Multiple copy integration

Although the defective *leu2*-*d* allele has been successfully used to increase plasmid copy number and protein production in *S. cerevisiae* it has not yet been tested for the same purpose in *K. phaffii*. In a previous work, attenuated *ADE1* and *ADE2* genes involved in adenine biosynthesis were used to develop a color-based system for the screening of multicopy integrants in *K. phaffii* [35]. However, this system is based on large plasmids and, in some cases, the effects of background transcription from vector sequences were responsible for the recovery of adenine prototrophy.

**Fig. 3:**
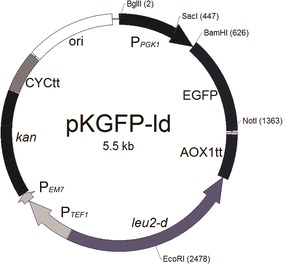
Physical map of pKGFP-ld expression vector for multiple copy integration in *K. phaffii.* leu2-d defective *LEU2* allele from *S. cerevisiae*. *P*_*PGK1*_ *and P*_*TEF1*_ promoters from the *K. phaffii PGK1* and *TEF1* genes, respectively, *P*_*EM7*_ synthetic *E. coli* promoter, *kan* G418/kanamycin resistance gene, *EGFP* enhanced green fluorescence gene, *CYCtt and AOX1tt* transcription termination sequences from *CYC1* and *AOX1* genes, respectively, *ori E. coli* origin of replication

We constructed an expression vector, pKGFP-ld (Fig. 3), carrying the *leu2*-*d* defective marker and an EGFP reporter construct under the control of the phosphoglycerate kinase 1 promoter (P_*PGK1*_). In order to circumvent the possibility of spurious marker expression, *leu2*-*d* was cloned in the opposite orientation of the other yeast promoters present on the vector (P_*PGK1*_ and P_*TEF1*_). Furthermore, the presence of the *kan* marker under the control of dual-promoters allows plasmid selection in *Escherichia coli* and may be used to confirm multicopy integration by plating transformed cells on media containing increasing concentrations of the antibiotic G418.

Seven days after transformation of *K. phaffii* M12 cells with pKGFP-ld, colonies of diverse sizes were observed on MD plates. Ten transformants representing colonies with different sizes were selected for further analysis. After a few passages on fresh MD plates four clones derived from the smallest colonies present on the original transformation did not grown upon replica plating and were removed from this study. We speculate that these abortive clones were transformed with a limited number of copies of the defective marker, thus they were unable to sustain growth under selective conditions.

**Fig. 4:**
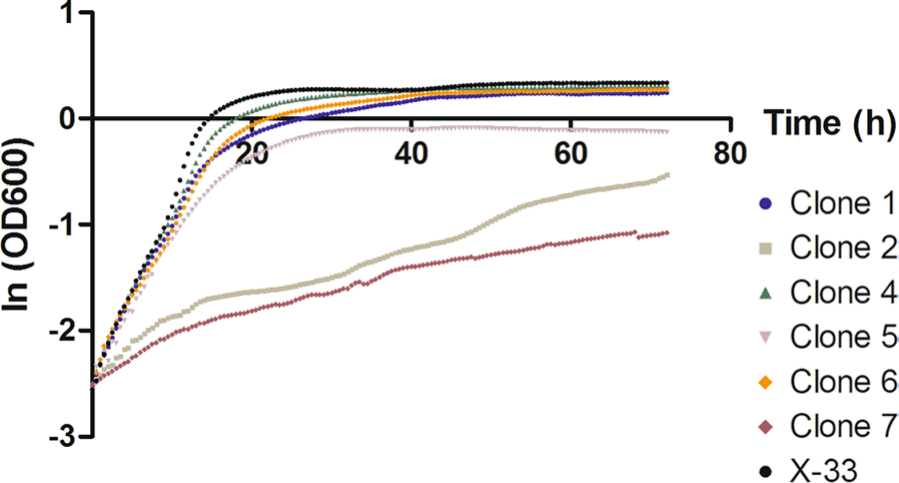
Growth kinetic of selected clones transformed with pKGFP-ld. Cells were grown on MD medium at 30 °C during 72 h. Growth was expressed as the natural logarithm of OD_600_ which was measured every 30 min. Initial OD_600_ = 0.08. *X-33 K. phaffii* wild-type strain

A growth kinetic analysis of the six remaining clones was performed on MD medium (Fig. 4). The calculated maximum growth rates (Table 1) showed that larger clones present on the original transformation plate (clones 1, 4, 5 and 6) exhibited a growth profile similar to that of *K. phaffii* X-33 whereas small sized colonies (clones 2 and 7), as expected, exhibited smaller growth rates. Analysis of variance followed by Tukey’s post-test showed significant difference in maximum growth rates presented in Table 1 for clones 2 and 7 when compared to X-33 (p < 0.05). Similar results were obtained when truncated *ADE1* and *ADE2* were used as selectable defective markers to transform *K. phaffii* [35]. In this case, multicopy clones were also identified as colonies with larger sizes. We hypothesized that, in order to compensate the poor transcription from *leu2*-*d*, cells would require additional integrated copies of defective marker to recover full prototrophy. This prompted us to determine the copy number of vectors integrated into the *K. phaffii* genome by Southern blot.

**Table 1:** Main features of the selected clones studied in this work

Clone	µ_max_ (h^−1^)	Vector copy number	EGFP production (fluorescence units)
1	0.1596 ± 0.0062	17	19215 ± 2388
2	0.0708 ± 0.0022	7	7705 ± 550
4	0.1614 ± 0.0027	20	23,688 ± 2152
5	0.1408 ± 0.0075	14	18,387 ± 1045
6	0.1650 ± 0.0078	17	20,074 ± 1738
7	0.0502 ± 0.0064	5	6711 ± 206
X-33	0.1707 ± 0.0069	0	ND

*µ*_*max*_ maximum growth rate, *ND* not determined

**Fig. 5:**
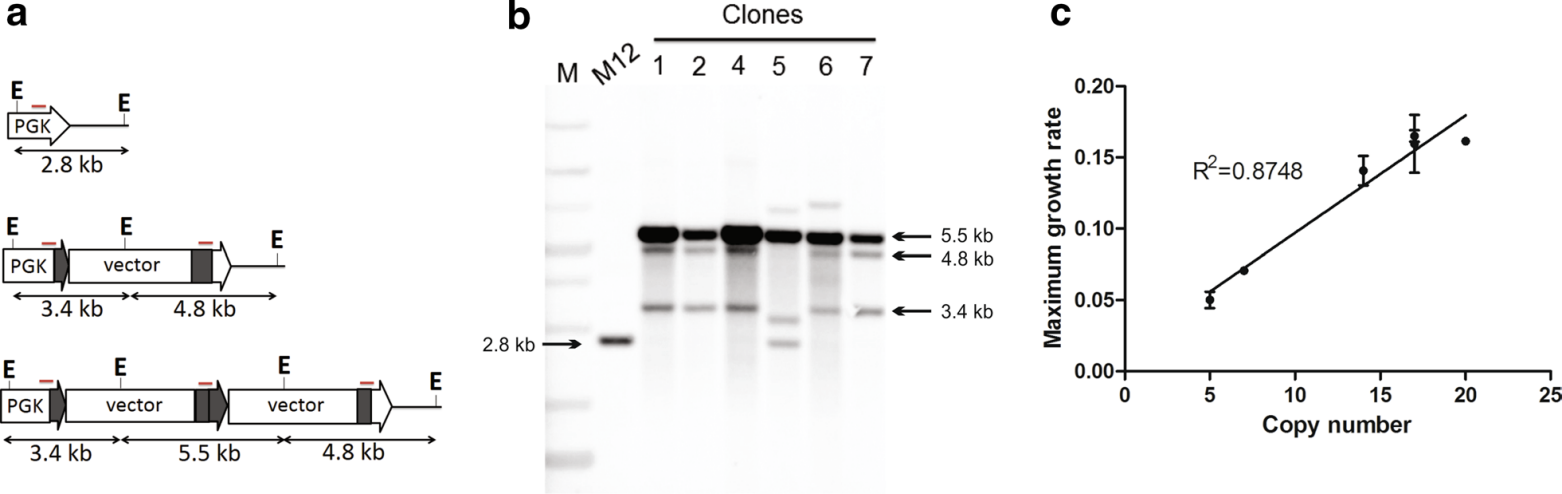
Vector copy number determination. **a** Schematic representation of genomic contexts and expected sizes of bands obtained after hybridization with a PGK probe (annealing positions are represented by a *red line*). *Dark grey areas* correspond to integrated P_*PGK1*_ sequences derived from vector. **b** Result of Southern blot analysis. **c** Correlation between copy number and maximum growth rates of selected clones. *Error bars* represent standard error of the mean. *M* O’GeneRuler 1 kb DNA ladder, *M12 K. phaffii leu2* strain, *E* EcoRI restriction site

Because pKGFP-ld was linearized with SacI, a restriction site within P_*PGK1*_ (Fig. 3), most events of integration would be primarily targeted to this *locus*. According to the schematic representation shown in Fig. 5a, if a single copy of the vector had integrated into the *PGK1 locus*, two fragments of 3.4 and 4.8 kb would be expected. Two or more integrated copies would yield an additional 5.5 kb fragment which would increase in intensity for each additional copy added. The results from the Southern blot analysis are shown in Fig. 5b. As expected, the M12 untransformed strain showed the 2.8 kb fragment, which corresponds to the intact *PGK1 locus*. All transformed clones showed the 5.5 kb fragment thus confirming the *in tandem* integration of at least two copies of the vector. The observation that clone 5 showed other bands (including the 2.8 kb fragment) which were not present in the other clones suggests that, in this particular clone, the vector had integrated in a different manner. This is not entirely unexpected since it has recently been shown that a transforming cassette can integrate into the *K. phaffii* genome in different cassette-to-cassette orientations and secondary recombination events may also occur [36]. Also, we cannot exclude the possibility of off-target integration events mediated by non-homologous end joining (NHEJ) which is the main repair system in filamentous fungi and higher eukaryotes [37]. Table 1 shows that that faster growing clones 1, 4, 5 and 6 showed the highest vector copy number (≥14 copies) as compared to slower growing clones 2 and 7 which had no more than 7 copies. Growth rate and copy number showed a linear correlation (R^2^ = 0.8748) (Fig. 5c). These results are in agreement with the prediction that faster growing clones would have more integrated copies of the defective marker.

### Genetic stability

Previous works have shown that single or low copy integrated messages are genetically stable in *K. phaffii* under different conditions [38, 39], however, few studies have focused on the integrity of multicopy clones. Since multicopy *K. phaffii* strains generally arise from multiple events of homologous recombination at the same *locus*, the integrated messages are typically repeated *in tandem*. The stability of such array may be compromised by excisional recombination which can “loop out” the genetic message under non-selective conditions [14], [40]. In order to test the stability of integrated pKGFP-ld, transformed cells were grown in non-selective medium (YPD) for 36 and 72 generations. The culture was transferred to fresh medium every 24 h to ensure that cells were in exponential phase throughout the experiment. After growth for 96 h or 144 h (36 and 72 generations, respectively), copy number of the selected clones was assessed by Southern blot analysis which showed that all clones maintained the original vector copy number (data not shown).

In a recent work*, S. cerevisiae* strains with multiple integrated cassettes bearing different defective auxotrophic markers also showed mitotic stability under prolonged nonselective conditions [41]. *S. cerevisiae* cells transformed with five or more copies of an integration vector conferring resistance to G418 and expressing *SUC2* (invertase) were very unstable during long-term culture in non-selective medium [42]. Likewise, when *K. phaffii* was transformed with a set of vectors containing sequentially increasing copies of porcine insulin precursor gene (PIP), both low and high copy strains were stable in serial culture in non-selective YPD medium. However, in high copy strains, loss of PIP cassettes was observed after 96 h of methanol induction [43].

Based on these previous results, it has been proposed that multicopy strains should be carefully evaluated for genetic stability especially under conditions of high expression or secretion [43]. In our work, since EGFP was produced intracellularly from a moderately strong *K. phaffii* promoter (P_*PGK1*_) [44], it is possible that the titers of this particular protein were not high enough to compromised cell growth as shown on Fig. 4, hence, genetic stability was observed.

### Correlation between copy number and protein production

**Fig. 6:**
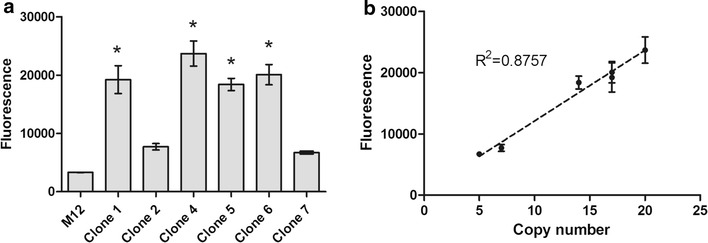
Intracellular EGFP production. **a** Flow cytometry analysis of the cells positive for EGFP production. **b** Correlation between copy number and EGFP production. *M12 leu2* strain. *Error bars* represent standard error of the mean. *Asterisks* indicate significant difference between the evaluated clone and the M12 control strain according to ANOVA followed by Tukey’s post-test (p < 0.05)

In order to determine the correlation between plasmid copy number and increased heterologous protein production, intracellular fluorescence emission of each selected clone was determined by flow cytometry. As shown in Fig. 6a, all selected clones exhibited fluorescence emission higher than the untransformed M12 strain. EGFP production was the highest in clones 1, 4, 5 and 6 which exhibited higher plasmid copy number, whereas moderate production was observed in clones 2 and 7 (Table 1). Analysis of variance followed by Tukey´s post-test showed significant difference in EGFP production in clones 1, 4, 5 and 6 when compared to M12 control strain (p < 0.05). The EGFP fluorescence from clones 2 and 7 was not high enough to result in statistically significant differences in EGFP production in comparison to M12 (p > 0.05). However, it is important to notice that the percentage of M12 cells producing EGFP (cells positive for EGFP) was less than 1%. As shown in Fig. 6b, we observed a linear correlation between vector copy number and EGFP production as measured by flow cytometry (R^2^ = 0.8757). It is expected that at a certain copy number the production of the heterologous protein might become detrimental to the cell; however, for the examined clones, we did not observe a decrease in cell viability nor in EGFP production which augmented linearly with up to 20 integrated copies. Similarly, an increase of up to eight copies of the hepatitis B surface antigen (HBsAg) gene showed a linear correlation with the concentration of mRNA and translated protein in *K. phaffii* [45].

However, the relationship between gene copy number and protein production is not always linear and in some cases it proved to be detrimental, especially for secreted proteins [6]. In another study involving EGFP, an increase of the secreted protein was observed with up to three copies but a decrease occurred with six copies [46]. When multicopy clones were used to produce intracellular human superoxide dismutase (hSOD) and secreted human serum albumin (HSA) a difference was observed in the correlation of gene copy number and productivity between non-secreted and secreted proteins [15]. The productivity of hSOD correlated linearly with gene copy number, while HSA productivity increased up to approximately 5–7 gene copies, and then decreased with higher copy numbers. *K. phaffii* strains secreting human trypsinogen under the control of the *AOX1* promoter presented a positive correlation between copy number and product yield from 1 to 2 copies per cell, and a negative correlation at 3 or more copies [47]. Upon overexpression, great part of the heterologous protein was retained in the insoluble fraction of the endoplasmic reticulum. From this studies it is clear that bottlenecks in the secretory pathway are to some extent responsible for the low productivity of some multicopy clones [6].

Since the effect of gene dosage may vary from one protein to another, it is not possible to define the optimal copy number for any specific heterologous gene which should be assessed on a case-by-case basis. However, by using the approach presented in this work one can easily obtain a panel of clones with different copy numbers to be screened for the desired application. Furthermore, we envision that this approach might be also applied in synthetic biology studies in which different doses of specific genes may be required. This could be rapidly achieved by transforming M12 with different plasmids bearing the *leu2*-*d* marker following a screening for the desired phenotype. Work is underway in our laboratory to test this new application.

## Conclusions

In this work, we proposed a simple approach to obtain *K. phaffii* clones containing multiple copies of a desired expression vector. Our genetic system is based on a *K. phaffii* strain auxotrophic for leucine which is transformed with an expression vector bearing a defective *leu2*-*d* marker. The main advantage of the approach proposed here is the ease in selecting multicopy clones, in our case this was based on colony size. This approach might serve as a first step in the construction of strains with higher productivity thus lowering the costs of industrial recombinant protein production.

## Methods

### Strains and growth conditions

*Komagataella phaffii* GS115 (*his4*) and X-33 (Invitrogen) were used as a source of template DNA to amplify *LEU2* and cell host to perform transformation with the disruption cassette, respectively. *K. phaffii* was routinely grown on YPD (1% yeast extract, 2% peptone and 2% glucose) at 28 °C. Solid medium was prepared by the addition of 2% agar. After transformation yeast cells were plated on YPD containing 300–500 µg mL^−1^ G418 or 150 µg mL^−1^ hygromycin B. Transformants were tested on MD [0.34% Yeast Nitrogen Base (YNB), 1% ammonium sulphate, 2% glucose, 0.4 µg mL^−1^ biotin and 2% agar] and buffered MD [MD with 100 mM potassium phosphate (pH 6.0)] supplemented or not with 0.04 or 0.08% leucine. For induction of heterologous gene expression from the P_*AOX1*_ promoter cells were grown in a medium containing 1% yeast extract, 2% peptone, 100 mM potassium phosphate (pH 6.0), 0.34% YNB, 1% ammonium sulphate, 0.4 µg mL^−1^ biotin supplied with 1% glycerol (BMGY medium) or 0.5% methanol (BMMY medium). When liquid medium was used, growth was carried out under agitation (200 rpm) in shake flasks with a volume at least 10 times greater than the volume of the medium.

Cloning procedures were carried out in *E. coli* XL10-gold (Stratagene, USA) which was cultivated in modified LB medium (0.5% yeast extract, 1% peptone and 1% NaCl) containing the appropriate antibiotic for selection of transformants (100 µg mL^−1^ ampicillin or 50 µg mL^−1^ kanamycin). Bacterial cells were grown at 37 °C with constant shaking (250 rpm). For solid medium, 1.5% agar was added.

### PCR

Phusion high-fidelity DNA polymerase (Finnzymes) was routinely used for PCR according to the instructions of the manufacturer. To amplify *LEU2*, Easy Taq DNA polymerase (LGC Bio, Brazil) was used in a final volume of 50 μL consisting of 0.2 mM dNTP, 0.2 μM each primer, 2 mM MgCl_2_, Easy Taq buffer 1X, 2 U polymerase and 1–5 ng template DNA. PCR involved an initial denaturation step at 96 °C for 3 min followed by 30 cycles of 60 s/94 °C, 1 min/60 °C, 2 min/72 °C and a final elongation step at 72 °C for 5 min.

### DNA manipulations

Plasmid extraction, electrophoretic analysis and other basic DNA manipulations were performed as described previously [48]. For DNA elution from agarose gels and for amplicon purification *Wizard SV Gel* and *PCR Clean*-*Up System* (Promega, USA) were used according to the manufacturer’s instructions, respectively. Genomic DNA was purified by using *Wizard Genomic DNA Purification Kit* (Promega, USA) using the manufacturer’s protocol.

### Construction of disruption cassette

First, *LEU2* was amplified by PCR from *K. phaffii* GS115 genome with primers PpLEU2-F1 and PpLEU2-R2 (Table 2) which introduce PvuII restriction sites at their 5´-ends. Primers were designed based on the published sequence of the *K. phaffii* chromosome 3 (accession # FR839630.1). The amplified 1.7 kb fragment included the *LEU2* coding region and ~300 bp of both upstream and downstream sequences. *LEU2* was cloned into pGEM-T easy (Promega) resulting in pGEM-LEU. This vector was digested with EcoRV to remove 375 bp of the coding region of *LEU2* (positions 405–750). To disrupt *LEU2*, first, a 1.7 kb fragment containing a *kan* expression cassette flanked by *lox*P sites was amplified from pPICKα [49] with primers ZeoBlas-F3 and ZeoBlas-R3 (Table 2). This amplicon was cloned into EcoRV-digested pGEM-LEU generating plasmid pLEUΔkan. Finally, the *leu2::kan* disruption cassette was released after digestion of pLEUΔkan with PvuII prior to yeast transformation.

**Table 2:** Primers used in this work

Primer	Sequence (5′→3′)*	Restriction site
PpLEU2-F1	CAGCTGAAGAGTCCAAGTCCAAG	PvuII
PpLEU2-R2	CAGCTGGTGCCATTGGTGGTACTGT	PvuII
TEF-2F	ATACCTAGGCCCCACACACCATAGCTTCAA	AvrII
TEF-2R	ATACCTAGGTTTGTAATTAAAACTTAGATTAGATTG	AvrII
ZeoBlas-F3	CGGATCCATAACTTCGTATAATGTATGCTATACGAAGTTATAGATCTCCCACACACCATAGCTTCAAAATG	BamHI and BglII
ZeoBlas-R3	CGGATCCATAACTTCGTATAGCATACATTATACGAAGTTATAGATCTAGCTTGCAAATTAAAGCCTTCGAG	BamHI and BglII
PpLEU2-EXT1	GAGGATAAGCTGGGAGACTATG	–
PpLEU2-EXT2	TCTGTTGCCTAAGACTGAGAGC	–
5-leud	GAGATCTATATATATTTCAAGGATATACCATTCTAATG	BglII
3-leud	GAGATCTGTTTCATGATTTTCTGTTACACC	BglII

* Restriction sites are underlined

### Marker excision

A vector based on pYRCre was constructed in order to promote marker excision in *K. phaffii*. Plasmid pYRCre was originally used to express the CreA recombinase in *S. cerevisiae* [49]. The P_*GAL1*_ promoter present in this vector was removed after XbaI digestion and replaced by a 441-bp fragment corresponding to the *S. cerevisiae* P_*TEF1*_ promoter which was obtained by PCR using primers TEF-1F and TEF-1R (Table 2). The amplicon was digested with AvrII and cloned into XbaI-digested pYRCre. The resulting vector, pYRCre2, was used to transform *K. phaffii* and selection was made on YPD plates containing hygromycin B. Transformants were incubated at 28 °C for 3 days to allow expression of CreA recombinase and then selected clones were transferred to an YPD plate for plasmid curing. Isolated colonies were replica plated on YPD plates containing G418 or hygromycin B to confirm the removal of *kan* marker and cure of pYRCre2, respectively.

### Construction of expression plasmid pGFP-L2

First, a vector constructed in our lab derived from pPIC9 (Invitrogen, USA) with the EGFP reporter gene under the control of the P_*AOX1*_ promoter was digested with EcoRV. This digestion removed the entire *HIS4* sequence, which was replaced by the *LEU2* gene obtained from pGEM-LEU after digestion with PvuII. The resulting vector, pPIC-LEU, was digested with BamHI and NotI to remove the EGFP gene which was fused in-frame to the α-factor secretion signal. This secretable version of EGFP was replaced by a 741 bp EGFP fragment from pEGFP-N3 (Clontech, USA) after digestion of this plasmid with the same enzymes. The resulting plasmid, which allows intracellular expression of EGFP, was named pGFP-L2. Before *K. phaffii* transformation pGFP-L2 was linearized with SacI to promote targeted integration to the P_*AOX1*_ *locus*.

### Construction of expression vector pKGFP-ld

The *leu2*-*d* allele was amplified by PCR from *S. cerevisiae* genome with primers 5- and 3-leud (Table 2). The amplified 1.4 kb fragment included the *LEU2* coding region with its transcription termination region and only 29 bp of its promoter [17]. The *leu2*-*d* amplicon was cloned into pBlueScript SK II (Agilent Technologies) and then liberated after BglII digestion for subcloning into BamHI-linearized pPICK2 [50] resulting in pK-ld vector. This vector was digested with SacI and NotI to remove the α-factor secretory sequence. This digestion also removed a 179 bp fragment from P_*PGK1*_ which was restored when the EGFP gene was cloned. The 916 bp fragment including the EGFP gene fused to the 179 bp fragment from the P_*PGK1*_ was obtained by digestion of pPICK-GFP [a vector derived from pPIC9 (Invitrogen) for intracellular expression of EGFP under the control of P_*PGK1*_] with SacI and NotI. Cloning of this 916 bp fragment into pK-ld resulted in pKGFP-ld vector. This vector was linearized with SacI to target integration to the *PGK1 locus*.

### Yeast transformation

*Komagataella phaffii* X-33 was transformed by electroporation following the protocol described in the *Pichia* Expression Kit (Invitrogen). Transformation with pYRCre2 was carried out as previously described for the auto-replicative pPICHOLI vector [51].

### Fluorescence microscopy

*Komagataella phaffii* cells expressing EGFP were grown in 5 mL BMGY for 16 h at 28 °C. After cell count, pelleted cells were re-suspended in 20 mL BMMY to a final OD_600_ of 0.3. The culture was incubated at 28 °C and methanol was added to a final concentration of 0.5%. After 24 h of induction cells were imaged in a Zeiss Axio Observer Z1 Inverted Fluorescence Microscope equipped with 63× NA 1.4 oil immersion objective and a cooled CCD camera to analyze EGFP fluorescence. The images were acquired with Zen2011 software (Zeiss) and manipulated with Microsoft Office Picture Manager or Adobe Photoshop.

### Growth kinetics

A fresh colony was inoculated in 500 µL of MD medium in a *deep*-*well* plate and incubated for 24 h at 30 °C and 200 rpm. The appropriate volume of this culture was inoculated in 100 µL of MD to an OD_600_ = 0.08 in a 96-well plate. Cell growth was performed on the Epoch Microplate Spectrophotometer (Biotek) by incubating at 30 °C under agitation of 300 rpm for 72 h. OD_600_ data was collected every 30 min. Three biological replicates were tested for each analyzed clone and the mean of the three values was presented. Natural logarithm of OD_600_ values was used to construct growth curves. Maximal growth rate was calculated from the slope of the linear section of these curves (up to eight hours growth).

### Southern blot analysis

Yeast cells were grown in 40 mL of MD medium at 30 °C under agitation during 24 h and the DNA was extracted using phenol–chloroform as previously described [48]. Aproximately 10 µg of genomic DNA were digested with EcoRI at 37 °C overnight. Digested DNA was applied in 0.8% agarose gel and then transferred to nitrocellulose membrane as described [48]. Probe labeling, hybridization and detection were made using the AlkPhos Direct Labeling and CDP-Star Detection System (GE Life Sciences) following especifications of the manufacturer. The probe used was a fragment of ~600 bp corresponding to the *PGK1* promoter obtained by digestion of pKGFP-ld with BglII and BamHI. The temperature for hybridization was 55 °C. Chemiluminescence was detected using the Amersham Imager 600 system (GE Life Sciences) and band intensity was measured with the use of the ImageQuant TL 8.1 software.

### Genetic stability testing

The stability of the heterologous DNA integrated into the yeast genome was tested in shake flasks after 36 and 72 generations. A fresh colony was grown in 10 mL YPD medium for 24 h at 30 °C and 200 rpm. Then, 400 µL of this pre-inoculum were inoculated in 40 mL YPD and incubated under the same conditions for 24 h. A 400 µL sample of the culture was transferred to a new flask with 40 mL YPD and incubated under the same conditions for 24 h. This procedure was repeated four more times for a total growth time of 144 h. After 96 h (36 generations) and 144 h (72 generations) genomic DNA was extracted and submitted to Southern blot analysis as described above.

### Flow cytometry

Yeast cells were grown in 5 mL of MD medium for 24 h at 30 °C and agitation. The required volume of each pre-inoculum was inoculated in 5 mL of MD to start the culture with an OD of 0.5. After 24 h of incubation at 30 °C under agitation cells were washed twice with PBS (137 mM NaCl, 2.7 mM KCl, 10 mM Na_2_HPO_4_ and 2 mM KH_2_PO_4_, pH 7.4) containing 0.5% Tween by centrifugation at 3000×*g* for 5 min at 4 °C. Cells were suspended in the required volume of PBS to obtain approximately 10^6^ cells mL^−1^. Cells were maintained at 4 °C until analysis with FACSVerse flow cytometer. All samples were collected with identical voltage parameters. Acquired data were analyzed using the FlowJo software. The gating strategy included: (a) gating on yeast cells on forward versus side scatter plots; (b) gating on single cells using forward scatter width versus forward scatter height plots and (c) selecting positive cells based on histograms from wild-type cells. Three biological replicates were tested for each analyzed clone and the mean of the three values is presented.

### Data analysis

Statistical analyses and figures were made on GraphPad Prims 5 software. ANOVA followed by Tukey’s post-test was applied. Error bars on graphics represent standard error of the mean.

## Additional file

**Additional file 1.** Disruption of *LEU2* in *K. phaffii* X-33. Panel A: Annealing positions of primers used to amplify *LEU2* and for diagnostic PCR. Expected sizes of amplicons are in the bottom of each figure. The *LEU2* coding sequences are in dark grey. Primers: 1 PpLEU2-F1, 2 PpLEU2-R2, 3 PpLEU2-EXT1, 4 PpLEU2-EXT2, 5 ZeoBlas-F3. Panel B: PCR analysis in 1% agarose gel electrophoresis stained with ethidium bromide. M: O’GeneRuler 1 kb DNA ladder; lanes 1, 3 and 5: PpLEU2-EXT1/PpLEU2-EXT2; lanes 2, 4 and 6: ZeoBlas-F3/PpLEU2-EXT2. X-33: wild-type strain with intact *LEU2* gene, LK: *leu2* strain disrupted with *kan* cassette, M12: strain obtained after marker removal with CreA recombinase.
