# Supplementary material for: Multicopy plasmid integration in Komagataella phaffii mediated by a defective auxotrophic marker
Source: Microb Cell Fact. 2017 Jun 8;16:99. doi: 10.1186/s12934-017-0715-8 (PMC5465527; doi:10.1186/s12934-017-0715-8)
Supplement: Supplementary file 1 — Additional file 1. Disruption of LEU2 in K. phaffii X-33. Panel A: Annealing positions of primers used to amplify LEU2 and for diagnostic PCR. Expected sizes of amplicons are in the bottom of each figure. The LEU2 coding sequences are in dark grey. Primers: 1 PpLEU2-F1, 2 PpLEU2-R2, 3 PpLEU2-EXT1, 4 PpLEU2-EXT2, 5 ZeoBlas-F3. Panel B: PCR analysis in 1% agarose gel electrophoresis stained with ethidium bromide. M: O’GeneRuler 1 kb DNA ladder; lanes 1, 3 and 5: PpLEU2-EXT1/PpLEU2-EXT2; lanes 2, 4 and 6: ZeoBlas-F3/PpLEU2-EXT2. X-33: wild-type strain with intact LEU2 gene, LK: leu2 strain disrupted with kan cassette, M12: strain obtained after marker removal with CreA recombinase. [file 12934_2017_715_MOESM1_ESM.docx]

**Construction of a *leu2 K. phaffii* strain**

Construction of a *leu2* strain was accomplished by a strategy involving the cloning of the *K. phaffii* *LEU2* gene by PCR followed by *in vitro* gene disruption with a *kan* expression cassette flanked by *lox*P sites for marker excision. A *leu2::kan* disruption cassette was used to transform *K. phaffii* X-33 cells following selection on plates containing G418. Twelve isolated colonies were replica plated onto MD medium supplemented or not with leucine. Seven colonies exhibited a Leu^-^ phenotype; one particular clone, named LK, was selected to further confirm *locus*-specific integration. This was performed by PCR using a combination of primers that annealed outside the region that was used to amplify *LEU2* and within the *kan* marker cassette (Fig. 1A). The first set of primers (PpLEU2-EXT1/ PpLEU2-EXT2) yielded amplicons of 1.9 and 3.3 kb with X-33 and LK, respectively (Fig. 1B, lanes 1 and 3). With the second combination of primers (ZeoBlas-F3/ PpLEU2-EXT2) no amplification was observed with X-33 while a 2.4 kb fragment was amplified from LK (Fig. 1B, lanes 2 and 4) thus confirming the successful disruption of *LEU2*.

The removal of the *kan* marker was accomplished by using the *Cre*/*lox*P system which has been for that purpose in several yeasts including *Saccharomyces cerevisiae*, *Kluyveromyces lactis*, *Schizosaccharomyces pombe* [1] and *K. phaffii* [2]. CreA is a 38 kDa bacteriophage-derived protein that catalyzes the recombination of two *lox*P sites. When *lox*P sites are in the same orientation the recombination mediated by CreA allows removal of the DNA sequence between the sites [3]. We constructed vector pYRCre2 to constitutively express the CreA recombinase in *K. phaffii*. This vector was derived from pYRCre [4] which contains the gene coding for CreA recombinase under control of the *S. cerevisiae* inducible P*_GAL1_* promoter. As *K. phaffii* does not metabolize galactose, P*_GAL1_* was replaced with the *S. cerevisiae* translation elongation factor 1-α promoter (P*_TEF1_*). After transformation of *K. phaffii* LK with pYRCre2 several colonies resistant to hygromycin B were obtained. As pYRCre2 is a replicative plasmid its loss occurs spontaneously under non-selective conditions [5]. After growth in YPD, isolated colonies were checked for plasmid loss and marker removal by replica plating on YPD containing hygromycin B or G418. A clone sensitive to both antibiotics was selected and denominated M12. To confirm the loss of the *kan* marker in M12, PCR was performed with the primer combination described above (Fig. 1A). A 1.7 kb-fragment was obtained with the first combination of primers thus indicating that the dominant marker had been removed; this was further confirmed with the second primer combination (Fig. 1B, lanes 5 and 6).





**Figure 1** Disruption of *LEU2* in *K. phaffii* X-33. Panel **A:** Annealing positions of primers used to amplify *LEU2* and for diagnostic PCR. Expected sizes of amplicons are in the bottom of each figure. The *LEU2* coding sequences are in dark grey. Primers: 1 - PpLEU2-F1; 2 - PpLEU2-R2; 3 - PpLEU2-EXT1; 4 - PpLEU2-EXT2; 5 - ZeoBlas-F3. Panel **B:** PCR analysis in 1% agarose gel electrophoresis stained with ethidium bromide. M: O’GeneRuler 1 kb DNA ladder; lanes 1, 3 and 5: PpLEU2-EXT1/PpLEU2-EXT2; lanes 2, 4 and 6: ZeoBlas-F3/PpLEU2-EXT2. X-33: wild-type strain with intact *LEU2* gene, LK: *leu2* strain disrupted with *kan* cassette, M12: strain obtained after marker removal with CreA recombinase.

**References**

1. Laplaza JM, Torres BR, Jin Y-S, Jeffries TW: **Sh ble and Cre adapted for functional genomics and metabolic engineering of *Pichia stipitis***. *Enzyme Microb Technol* 2006, **38**:741–747.

2. Pan R, Zhang J, Shen W-L, Tao Z-Q, Li S-P, Yan X: **Sequential deletion of *Pichia pastoris* genes by a self-excisable cassette**. *FEMS Yeast Res* 2011, **11**:292–298.

3. Marx H, Mattanovich D, Sauer M: **Overexpression of the riboflavin biosynthetic pathway in *Pichia pastoris***. *Microb Cell Fact* 2008, **7**:23–33.

4. Reis VCB, Nicola AM, de Souza Oliveira Neto O, Batista VDF, de Moraes LMP, Torres FAG: **Genetic characterization and construction of an auxotrophic strain of *Saccharomyces cerevisiae* JP1, a Brazilian industrial yeast strain for bioethanol production**. *J Ind Microbiol Biotechnol* 2012, **39**:1673–1683.

5. Da Silva NA, Bailey JE: **Influence of plasmid origin and promoter strength in fermentations of recombinant yeast**. *Biotechnol Bioeng* 1991, **37**:318–324.
